# Pregnancy at high altitude: the challenge of hypoxia

**DOI:** 10.1098/rstb.2024.0167

**Published:** 2025-08-21

**Authors:** Graham J. Burton, Lorna G. Moore, Dino A. Giussani, Andrew J. Murray

**Affiliations:** ^1^ Department of Physiology, Development and Neuroscience, University of Cambridge, Cambridge CB2 3EL, UK; ^2^ University of Colorado Anschutz Medical Campus, Aurora, CO 80045, USA

**Keywords:** pregnancy, hypoxia, high altitude

Life at high altitude poses many challenges to human and animal populations alike, of which the most fundamental and universal is the reduced availability of oxygen. It is estimated that over 81 million people worldwide live at altitudes higher than 2500 m, the level at which normal physiological adaptations are unable to fully compensate for the reduced oxygen availability in the atmosphere. Exposure to hypobaric hypoxia has particularly profound effects on reproduction. This is especially true for recent migrants to high altitude, who may suffer infertility, have low-birthweight babies, and have a significantly higher risk of complications of pregnancy. By contrast, indigenous populations who have lived at elevation for many generations are protected to some extent by genetic and physiological adaptations. A Scientific Discussion Meeting was held in Cambridge, UK on 16−17 September 2024 to explore the challenges of life at high altitude for pregnancy, analysing the impact on the various steps in the maternal–fetal supply line that conveys oxygen and nutrients essential for fetal growth and how adaptations can mitigate the problems. Although the focus was primarily on the human, attention was paid to animal models, both natural and experimental. Finally, strategies for improving fetal oxygenation in pregnancies complicated by hypoxia at sea level were considered, along with the long-term impact of intrauterine hypoxia on lifelong health.

The contributions from anthropologists, epidemiologists, geneticists, physiologists, clinicians and developmental biologists brought together in this issue provide unique in-depth coverage of insights gained from this fascinating experiment-of-nature.

In the first paper, Lorna Moore [1] sets the scene, emphasizing the interactions between the three principal players during a pregnancy, the mother, the placenta and the fetus. Historically, most research has focused on fetal adaptations to pregnancy at high altitude, although more recently attention has turned towards the placenta. By comparison, few studies have investigated maternal responses, which is surprising given the importance of uteroplacental blood flow for fetal growth. Current studies employing ultrasonography to measure blood flows and blood gas measurements in the maternal and fetal circulations at delivery are beginning to fill this knowledge gap.

Emma Mitchell-Sparke, Catherine Aiken [2] and colleagues next consider the incidence of complications of pregnancy at altitude. They stress the difficulties in comparing data from different studies owing to the clinical diagnostic criteria used and confounders such as socio-economic status, urban versus rural locations, the healthcare system operating, and the age and ethnicity of the participants. Hence, data on the incidence of pre-eclampsia have proved conflicting. A large epidemiological study based on publicly available records in Ecuador showed no significant increase at high altitude, although the incidence of gestational hypertension was higher when the data were adjusted for all confounders. However, an increased risk was noted at the extremes of maternal age and among women with publicly funded care.

Genetic variants selected for in indigenous populations are discussed by Wanjun Gu, Tatum Simonson and colleagues [3]. They explain how epigenetic changes can occur rapidly in response to extreme environmental conditions and then become fixed within a population. In Tibetans, Denisovan-enriched mutations in the hypoxia-response pathway blunt the increase in haemoglobin concentration usually seen in non-indigenous migrants at high altitude. Consequently, blood viscosity is maintained at sea-level values, permitting normal cardiovascular function and facilitating uteroplacental oxygen delivery.

Sara Hillman and Padma Dolma [4] next report on a Himalayan population in Leh, Ladakh, resident at over 3500 m. Women who delivered a low-birthweight baby were older and lighter and had smaller maternal uterine artery diameters at 18−22 weeks gestation than those delivering babies in the normal range. The difference in arterial diameters was not maintained in a low-altitude control cohort. Genetic analysis of Ladakh babies replicated previously identified polymorphisms related to metabolic, skeletal and height genes.

Xiang-Qun Hu, Lubo Zhang and colleagues [5] provide a broad overview of factors regulating the tone of the uterine arteries and how they may adapt or be maladaptive at high altitude. These range from cytokines, hormones and growth factors, through epigenetic mechanisms involving microRNAs, oxidative and endoplasmic reticulum stresses, to nitric oxide and ion channels. Both structural and functional changes in the arteries in response to these factors are considered.

In the next presentation, Ramón Lorca [6] expands on the role of adenosine monophosphate-activated protein kinase (AMPK) as a vasodilator of the myometrial arteries. AMPK-dependent vasodilator responses are amplified in uterine arteries from mice exposed to hypoxia during pregnancy and in myometrial arteries from pregnant women at high altitude, while the opposite is true in arteries from women with low-birthweight pregnancies. AMPK activators may therefore be potential therapeutic agents to improve uteroplacental blood flow, although further research is needed to minimize off-target effects that may adversely affect fetal metabolism.

Colleen Julian [7] emphasizes the need to look beyond reduced uteroplacental blood flow as a cause of the lower birthweight at high altitude, pointing out that uterine oxygen supply still exceeds combined fetal and placental consumption. She proposes that the AMPK signalling pathway has broader roles, coordinating uteroplacental perfusion and fetoplacental metabolism through its actions as a vasodilator and a regulator of cellular energy homeostasis. Appreciating this crosstalk should increase understanding of how hypoxia influences fetal growth.

Picking up on energy homeostasis, placental mitochondrial respiration is the focus of the presentation from Andrew Murray, Jenna Armstrong [8] and Katie O’Brien. The placenta accounts for 30–40% of fetoplacental oxygen consumption, and they discuss how switching substrate usage from oxygen-demanding fatty acids towards glucose may help preserve oxygen availability for the fetus. However, at the same time, it could contribute towards fetal hypoglycaemia and low birthweight. Reducing mitochondrial oxidative phosphorylation at high altitude will also help to lower placental oxidative stress, a characteristic feature of pre-eclampsia.

Moving on to effects on the embryo, Dino Giussani [9] presents data from the chicken embryo, a model system that has the unique advantage of being independent of maternal cardiovascular changes and the placenta. Embryonic weight at hatching is reduced following incubation at high altitude compared with incubation at sea level, and this effect is mitigated by high-altitude ancestry, as in the human situation. Therefore, these findings emphasize that adaptations in individuals of prolonged highland residence ancestry may not be just at the level of the uteroplacental circulations. Furthermore, incubation at high altitude programmes the chicks to develop cardiovascular disease in adult life, providing a very tractable model system for investigating the mechanistic links between chronic fetal hypoxia and heart disease risk in humans.

Trond Michelsen [10] and colleagues have developed a four-vessel sampling system for aspirating blood from the uterine and umbilical arteries and veins at the time of Caesarean section, allowing precise maternal–fetal comparisons of blood gases and metabolites. They show that maternal body mass index (BMI) is negatively associated with fetal insulin sensitivity, influencing fetal metabolism, oxygen uptake and acid–base status. These findings underscore the significant role of maternal BMI in fetal health during pregnancy.

Irene Cetin and Isabella Abati [11] discuss metabolic adaptations of the human fetus to a reduced oxygen supply. They show that in normal pregnancies, oxygen delivery to the fetus on a per kilogram basis is remarkably consistent with that of other animal species. By contrast, low-birthweight babies show a strikingly reduced uptake of oxygen and glucose but the glucose/oxygen quotient is significantly increased in pregnancies complicated by fetal growth restriction. They conclude that in hypoxic pregnancies, the human fetus triggers adaptive mechanisms to reduce its metabolic rate, matching the proportion of substrate consumption relative to oxygen delivery as a survival strategy.

In a similar vein, Hannah Kyllo and Stephanie Wesolowski [12] present metabolic data generated from sheep models of chronic placental insufficiency and hypoxia. They explore how adaptations related to oxygen, glucose, lactate, pyruvate and amino acid flux may function to maintain rates of oxidative metabolism that enable the fetus to defend its growth rate. In the face of constraints in the supply of oxygen and nutrients across gestation, they speculate that growth-restricted fetuses develop a lower metabolic set point with these metabolic adaptations to ensure survival *in utero*.

Anibal Llanos [13] and colleagues next present data on metabolic adaptations in llamas breeding on the Andean Altiplano. Whereas lowland fetal mammals, including sheep, respond to hypoxia with a fetal brain-sparing effect, the llama fetus shows no increase in cerebral blood flow. Instead, there are reductions in cerebral oxygen consumption, Na-K-ATPase activity, Na-channel expression and temperature, which combine to protect against brain injury. Furthermore, newborn llamas do not experience pulmonary hypertension at high altitude as they show enhanced HO–CO signalling that offsets the pulmonary constrictor effects of hypoxia.

The effects on the lifelong health of the offspring caused by maternal exposure to hypobaric hypoxia during pregnancy are explored by Emilio Herrera and colleagues [14], who review research based on their sheep model. They describe short- and long-lasting changes in the systemic and pulmonary circulations, such as pulmonary hypoxic vasoconstriction, and the molecular mechanisms involved, including nitric oxide and carbon monoxide signalling and the role of oxidative stress.

Lifelong effects are considered further by Hayat Baba, Gina Galli and colleagues [15], who focus on the effects of prenatal hypoxia on the developing heart and their interactions with post-natal lifestyle choices. Male rats exposed to hypoxia during late pregnancy showed pulmonary hypertension and thickening of the right ventricular wall, whereas those exposed to normoxia followed by a high-salt diet post-natally displayed systemic hypertension and left ventricular thickening. Combining the two stressors resulted in a combination of the two phenotypes, suggesting that post-natal diet can worsen the effects of intrauterine hypoxia.

Finally, Kimberly Botting-Lawford and colleagues [16] present encouraging results showing that adverse cardiovascular effects in the offspring caused by exposure to chronic hypoxia while *in utero* can be mitigated by administration of antioxidants. These authors emphasize the delicate balance that must be struck between preserving the redistribution of blood to the brain, the ‘fetal brain-sparing effect’, which is mediated by the superoxide anion (O₂·⁻) and preventing the indiscriminate damage to biomolecules that the same species can cause. Targeting the antioxidants to the mitochondria, the principal site of reactive oxygen species generation, by use of MitoQ appears to have great potential as a possible therapeutic agent.

We are very grateful to the Royal Society for sponsoring the meeting, to all the invited speakers, session chairs and delegates for contributing to its success, and to Helen Eaton for her editorial help in compiling this issue.

## Data Availability

This article has no additional data.
